# Supplementary material for: Type I Interferon Signaling Controls Gammaherpesvirus Latency In Vivo
Source: Pathogens. 2022 Dec 17;11(12):1554. doi: 10.3390/pathogens11121554 (PMC9787724; doi:10.3390/pathogens11121554)
Supplement: Supplementary file 1 [file pathogens-11-01554-s001.zip › pathogens-2029296-supplementary.pdf]

Type I Interferon Signaling Controls Gammaherpesvirus Latency *in vivo*

Johannes Schwerk, Lucas Kemper, Kendra A. Bussey, Stefan Lienenklaus, Siegfried Weiss, Luka Čičin-Šain, Andrea Kröger, Ulrich Kalinke, Christopher M. Collins, Samuel H. Speck, Martin Messerle, Dagmar Wirth, Melanie M. Brinkmann, Hansjörg Hauser and Mario Köster

Figure S1

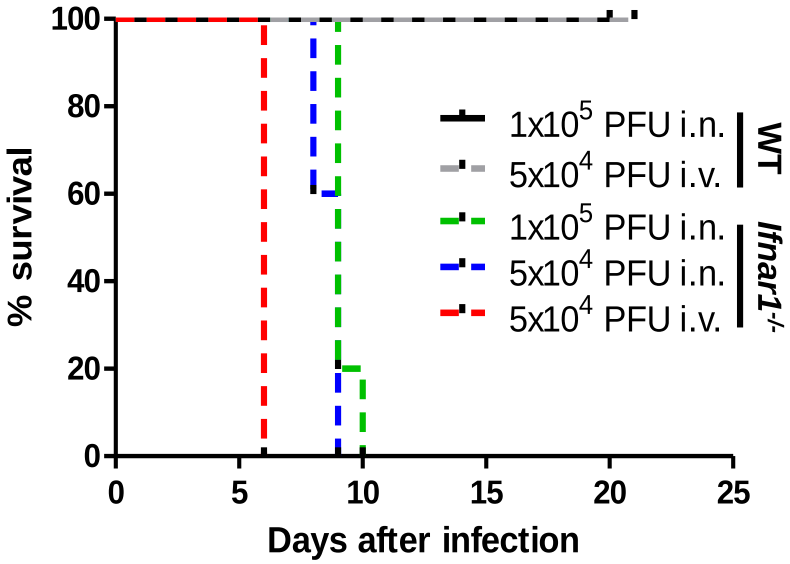

Figure S1. Type I IFN signaling is essential for survival of MHV-68 infection. Survival of WT and *Ifnar1*$^{-/-}$ mice upon intranasal (i.n.) and intravenous (i.v.) infection with indicated doses of MHV-68 WUMS (n=4-5).

Figure S2

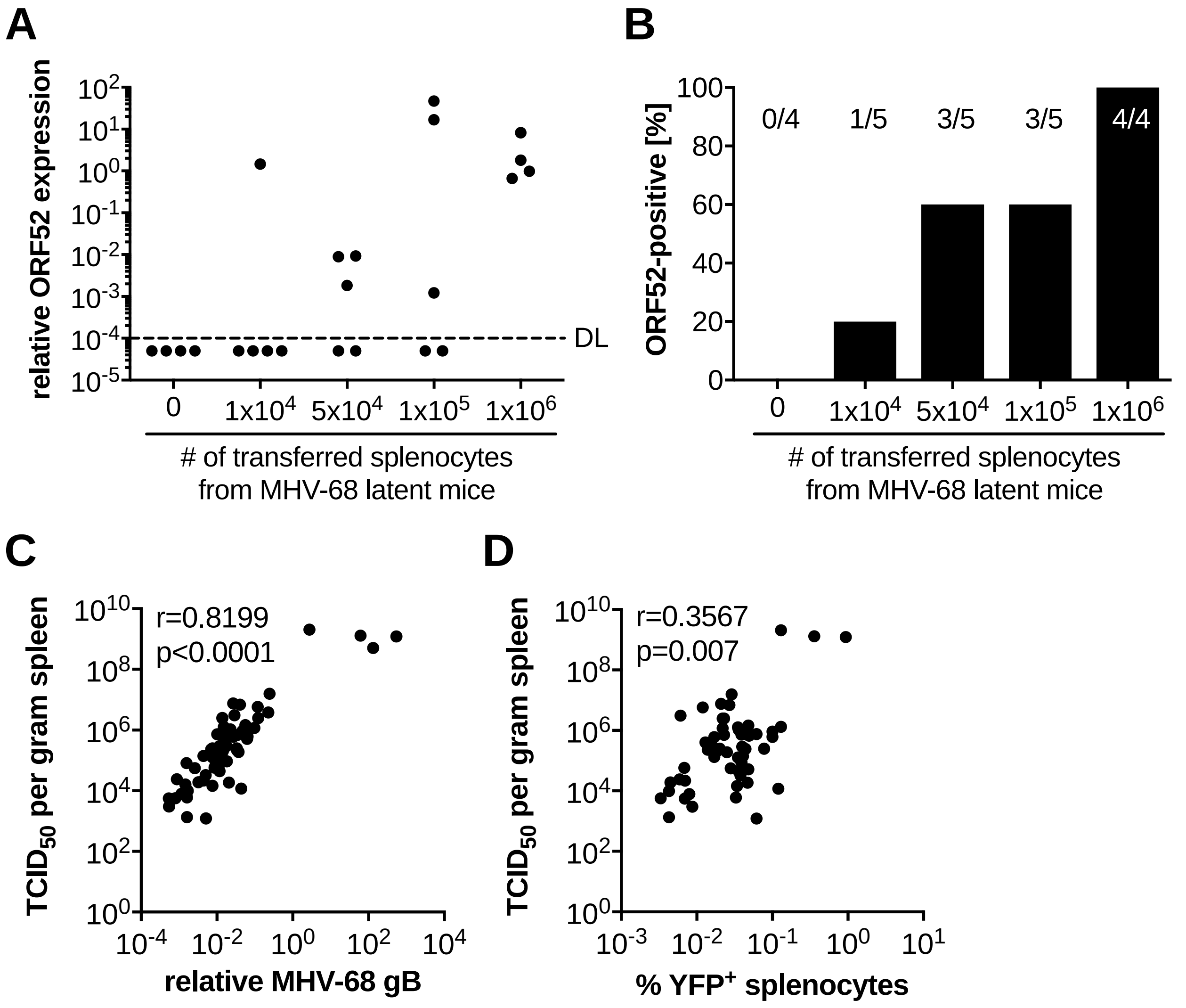

Figure S2. *In vivo* reactivation efficiency and validation of MHV-68 gB DNA and YFP⁺ cells as readout for recoverable infectious virus. (A) Indicated amounts of splenocytes from latently infected donor mice, supplemented with uninfected splenocytes to a total number of $1x10^7$ cells, were adoptively transferred into *Ifnar1*$^{-/-}$ recipient mice. Spleens were isolated from recipient mice 12 days after transfer and analysed for viral ORF52 expression by qPCR (n=4-5). **(B)** Ratio of reactivation-positive mice relative to the amount of adoptively transferred splenocytes from latently infected donor mice. **(C, D)** MHV-68 genome load (gB) and frequency of YFP^+ cells in spleens was correlated to recoverable infectious virus. The Spearman correlation coefficient (r) was determined by pooling data of several experiments (n=56-63).

Figure S3

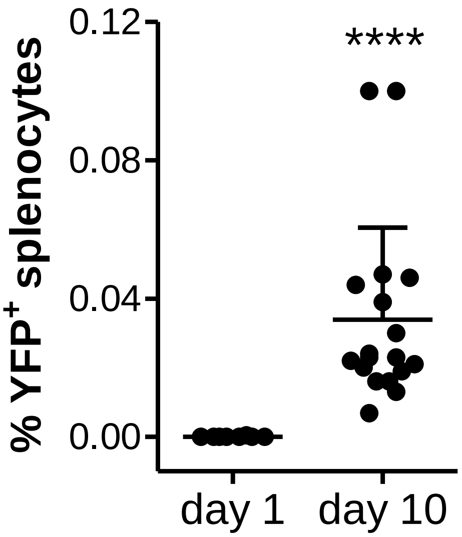

Figure S3. Increase of MHV-68 infected cells between day 1 and day 10 after adoptive transfer of latently infected splenocytes. Splenocytes from MHV-68 H2bYFP latently infected tamoxifen-inducible *Ifnar1*$^{-/-}$ mice (R26CreERT2 x *Ifnar1*$^{fl/fl}$) were isolated and adoptively transferred into conventional *Ifnar1*$^{-/-}$ recipient mice (10^7 cells/mouse). The frequency of YFP$^+$ splenocytes was determined by flow cytometry at days 1 and 10 after adoptive transfer. The graph compares the frequency of YFP$^+$ cells in spleens of recipient mice at day 1 and day 10 (see Figure 3F) after adoptive transfer without tamoxifen treatment of donor cells (day 1, n=8; day 10, n=18; mean ± SD). *P* value was calculated by Mann-Whitney's U test (****$P < 0.0001$).

Figure S4

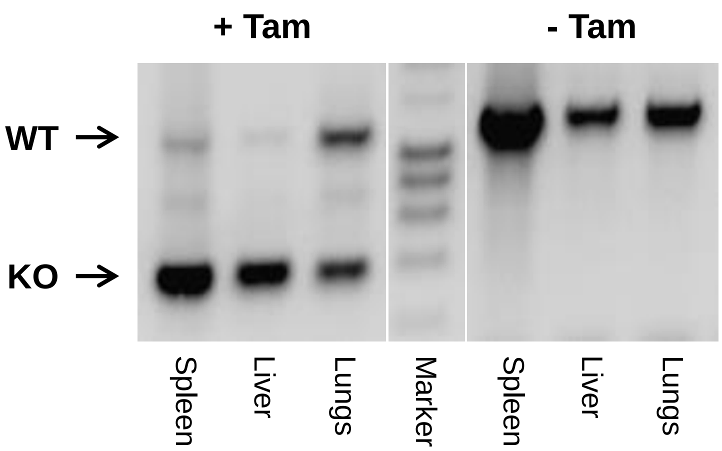

Figure S4. Tamoxifen-inducible knockout of *Ifnar1*. Novel R26CreERT2 x *Ifnar1*$^{fl/fl}$ mice were treated by oral administration of a single dose of 2 mg tamoxifen (+Tam). Mice were sacrificed 2 days later and genomic DNA was isolated from different tissue and subjected to PCR in order to verify efficiency and control of Cre-mediated recombination of *Ifnar1*. Untreated mice (-Tam) served as controls.
